# Early-life exposure to humidifier disinfectant determines the prognosis of lung function in children

**DOI:** 10.1186/s12890-019-1028-y

**Published:** 2019-12-23

**Authors:** Hyun-Ju Cho, So Yeon Lee, Donguk Park, Seung-Hun Ryu, Jisun Yoon, Sungsu Jung, Eun Lee, Song-I Yang, Soo-Jong Hong

**Affiliations:** 1 Department of Pediatrics, International St. Mary’s hospital, Catholic Kwandong University College of Medicine, Incheon, Republic of Korea; 2 Department of Pediatrics, Childhood Asthma Atopy Center, Environmental Health Center, Asan Medical Center, University of Ulsan College of Medicine, Seoul, Republic of Korea; 3 Department of Environmental Health, Korea National Open University, Seoul, Republic of Korea; 4 Department of Environmental Health Sciences, Graduate School of Public Health, Seoul National University, Seoul, Republic of Korea; 5 Department of Pediatrics, Mediplex Sejong Hospital, Incheon, Republic of Korea; 6 Department of Pediatrics, Pusan National University Yangsan Hospital, Yangsan, Republic of Korea; 7 Department of Pediatrics, Chonnam National University Hospital, Chonnam National University Medical School, Gwangju, Republic of Korea; 8 Department of Pediatrics, Hallym University Sacred Heart Hospital, Hallym University College of Medicine, Anyang, South Korea

**Keywords:** Chemical airborne exposure, Humidifier disinfectants, Lung function, Diffusing capacity, Children

## Abstract

**Background:**

Use of humidifier disinfectants (HD) at home leads to chemical airborne exposure, causing HD associated lung injury (HDLI) with high mortality. However, the lung function in children diagnosed with HDLI is not well studied. We investigated the effect of HD exposure on lung function, prognosis, and exposure characteristics associated with the lung function phenotype in children.

**Methods:**

Eighty-one children diagnosed with HDLI in a nationwide cohort were tested for spirometry and diffusing capacity of the lung for carbon monoxide (DLco) from July 2013 and followed up with at five time points over 2 years. The results were compared with 122 children without HD exposure as controls. Home investigation and questionnaire analysis were conducted to assess HD inhalation exposure.

**Results:**

HDLI survivor’s mean percent of predicted forced expiratory volume in 1 s (FEV_1_), forced vital capacity (FVC), and corrected DLco were significantly lower compared with the control group. On longitudinal assessment, FVC was within the normal range, but flattened, and spirometry showed a predominantly restrictive pattern. Corrected DLco did not normalize above 80% despite increasing age. The persistently low phenotype of lung function was associated with initial exposure age, especially less than 12 months of age. Higher density HD exposure during sleep and close distance between the bed and the humidifier were significantly associated with persistently low corrected DLco.

**Conclusions:**

HD exposure affects prolonged decrement in lung function, especially DLco, particularly among children who are exposed within the first year of life. These results suggested that early-life HD exposure determines long-term prognosis of lung function in children.

## Background

Humidifier disinfectants (HD), as household chemicals, have been used to prevent microbial growth in humidifier water tanks since 1994 in South Korea and contain various concentrations of chemicals, including oligoethoxyethyl-guanidinium chloride (PGH), polyhexamethylene-guanidine phosphate (PHMG), and chloromethylisothiazol/methyl-isothiazol (CMIT/MIT). These chemicals have been widely used in either industrial or various consumer products, including cosmetics, as biocides [1, 2]. However, HD inhalation from a humidifier’s aerosolizer was finally identified as a respiratory toxicant [3, 4] after an epidemic outbreak of idiopathic childhood interstitial lung disease (chILD) characterized by spontaneous air leak, rapid progression, and high mortality among immunocompetent children in South Korea in 2006 [5, 6]. Thereby, the novel forms of HD associated lung injury (HDLI) were defined in 2011, and HDLI has been added in the chILD classification of exposure-related ILD, worldwide [7, 8]. HD use in early-life was also recently found to independently increase the risk of childhood asthma [9].

Many chemical airborne exposures are associated with a decrement in lung function in adults, including in cases of large man-made disasters, such as the 1984 Bhopal Union Carbide methyl isocyanate gas release, the 2005 chlorine gas release from the Graniteville train accident, and current 2006 epidemic HD exposure (Additional file 1: Table S1). HD inhalation causing HDLI has been found to be characterized by restrictive lung defect in adults [10, 11]. Despite the associations observed in HDLI adults, the potential effects and the long-term consequences of HDLI children on lung function have not been explored, although children’s lungs are more susceptible to airborne exposures than those of adults [12, 13]. Even in case of the other chemical-related fatalities, no study on lung function in children has been reported. This continues to be a significant public health issue because there is no reference on chemical-related health crises of children, although thousands of toxic chemicals are used worldwide. Therefore, the characteristics of lung function and the presence of associated risk factors after HD inhalation are important in determining whether lung function can improve in the future in HDLI children. Furthermore, most studies in adults about chemical inhalants have been quantified by spirometric indexes, whereas less is known about diffusing capacity of the lung for carbon monoxide (DLco), which is important to evaluate the alteration of small airways after chemical exposure. Study of changes in DLco over time is useful to follow the course of disease [14].

Therefore, we investigated the effect of inhalation exposure to HD on lung function using both spirometry and DLco. In addition, we studied the long-term prognosis during childhood, which suggested the lung function phenotype and exposure characteristics associated with the phenotype.

## Methods

### Study design and participants

#### HDLI survivors

The Korean Government completed the third round of investigation from July 2013 to August 2016 in individuals with lung disease presumed to be related to HD exposure. Complete medical records, including demographics, laboratory findings, chest radiographs, and computed tomography scans and/or biopsy were collected. A Lung Injury Investigation Committee was formed by pulmonologists, radiologists, and pathologists to confirm the diagnosis of HDLI on the basis of clinical, radiological, and pathological findings and HD exposure assessment. Pulmonologists, radiologists, and pathologists then independently assessed the records of each case for relevant features based on diagnostic criteria and classified the features in each area into definite, probable, possible, or unlikely groups [15]. Thus, independent assessments of clinical features, radiographic findings, and pathology were combined to obtain an overall assessment for each case. Subsequently, the class of HDLI (definite, probable, possible, or unlikely) was determined. This study analyzed subjects with definite or probable diagnoses of HDLI. The subjects were prospectively followed in a nationwide cohort study and underwent spirometry and DLco. We excluded pulmonary function test (PFT) results that were repeated less than 3 months after the examination.

#### Control group

To compare the PFT between HDLI survivors and healthy Korean children, children as controls were recruited from the outpatients of Asan Medical Center from June 2016 through March 2018. All subjects were born at full term and had no history of exposure to HD. Subjects had no other diseases known to influence the respiratory system, either directly or indirectly.

### Pulmonary function testing

Spirometry and DLco were performed using Vmax229D instrument (SensorMedics, Yorba Linda, CA, USA) according to the American Thoracic Society (ATS)/European Respiratory Society (ERS) recommendations and extrapolated to ages over 7 years [14, 16]. Reference equations from a study by Morris et al. were used for spirometric parameters and from Polgar et al. for DLco [17]. We considered a percent predicted forced vital capacity (FVC) less than 80% to be abnormal [18]. Patterns of spirometry abnormality were defined as follows: normal [FEV_1_/FVC ≥ lower limit of normal (LLN) and FVC ≥ LLN], restrictive, (FEV_1_/FVC > LLN and FVC < LLN), obstructive (FEV_1_/FVC < LLN, FVC > LLN and FEV_1_ < LLN), and mixed (FEV_1_/FVC < LLN and FVC < LLN) [19]. Corrected DLco (adjusted for Hb, as recommended by the ATS/ERS [14]) less than 80% was defined as a significant diffusion defect [20–22]. The severity of the corrected DLco of 60 to 80%, 40 to 60%, and less than 40% were classified as mild, moderate, and severe abnormalities, respectively [22].

### Exposure assessment to humidifier disinfectant

The methods used for investigating use characteristics of HD based on personal interviews and home investigations have been described in Text S1 (“exposure assessment to HD”). These systematic and transparent exposure assessment approaches used to estimate the probability, frequency, and intensity of exposure to HD have been used in many published epidemiologic analyses to examine the association between HD exposure and clinically diagnosed lung disease [15, 23].

### Statistical analysis

Lung function outcomes were expressed as means with standard errors of the estimated mean (SE). The Chi-square or Student t-test was used to determine group differences on variables, as appropriate. Associations between initial exposure age and lung function were analyzed with the Cox proportional hazard regression model adjusting for height and weight at PFT performance, HD strands, and total months of HD use and were reported as hazard ratios (HR) with 95% confidence intervals (CI). Analyses were performed using SPSS, version 24.0 (SPSS Inc., Chicago, IL, USA). Figures were generated using GraphPad Prism 5.0 (Graphpad, San Diego, CA, USA). *P* < 0.05 was considered as statistically significant.

## Results

### Follow-up and demographics of the study subjects

Additional file 2: Figure S1 depicts the progression of participation in the Korean government survey. Final criteria were met by 81 HDLI survivors and 122 controls. Furthermore, 81 HDLI survivors were followed up in five time points with a mean interval of 7.2 months for spirometry and 6.1 months for DLco over approximately 2 years. Table 1 summarizes the study population characteristics, and no discernable demographic differences were observed between HDLI survivors and controls.

**Table 1:** Demographics of study population

	HDLI survivors (*n* = 81)	Control group (*n* = 122)	*P*-value
	mean or n (SD or %)	mean or n (SD or %)	
Age at spirometry (years)	8.6 (2.2)	8.7 (1.8)	0.744
Age at DLco (years)	9.3 (2.6)	8.7 (1.8)	0.085
Sex			
Boy	47 (58.0)	81 (66.4)	0.226
Girl	34 (42.0)	41 (33.6)	
Height (cm) at spirometry	133.5 (15.2)	133.7 (12.4)	0.925
Weight (kg) at spirometry	32.6 (14.0)	32.7 (11.5)	0.952
BMI (kg/m^2^) at spirometry	17.5 (3.5)	17.8 (2.9)	0.542
Height (cm) at DLco	137.6 (16.1)	133.7 (12.4)	0.064
Weight (kg) at DLco	35.9 (15.9)	32.7 (11.5)	0.123
BMI (kg/m^2^) at DLco	18.1 (3.8)	17.8 (2.9)	0.468

Data are presented as mean (standard deviation) or frequency (percentage) of subjects. *P* values for the comparison between HDLI survivors and controls calculated with the use of the Pearson chi-square test or the Student t test, as appropriate. HDLI, HD associated lung injury; N, number; SD, standard deviation; DLco, diffusing capacity of the lung for carbon monoxide; BMI, body mass index

### Comparison of pulmonary function test results: HDLI survivors vs. control subjects

HDLI survivors had a significantly reduced mean percent of predicted FVC, FEV_1_, DLco, and corrected DLco compared with controls in both sexes of children aged over 7 years (Table 2, total group). To determine the acceptable age to apply the normal criteria for corrected DLco (> 80%) in children, as less than 80% of corrected DLco was observed among girls over 7 years of age used a controls and since we had no reference in Asian children, we further analyzed the findings in children over 8 years and over 9 years of age (Table 2, subgroup). Controls of both sexes over 8 years of age showed a greater than 80% in corrected DLco, and the result was more pronounced in girls over 9 years of age. The age-related difference could be accounted for by the greater somatic size in boys than in girls [24].

**Table 2:** Comparison of PFT in HDLI survivors vs. control subjects

	HDLI survivors		Control group		*P*-value
	*n*	mean (SE)	*n*	mean (SE)	
A. Boy, Over 7 years					
FVC (% of pred)	47	87.4 (2.2)	75	98.5 (1.2)	< 0.001
FEV_1_ (% of pred)	47	86.0 (2.0)	75	96.7 (1.3)	< 0.001
FEV_1_/FVC	47	89.1 (0.7)	75	89.0 (0.5)	0.907
cor. DLco (% of pred)	47	74.0 (2.4)	81	87.1 (1.3)	< 0.001
Girl, above 7 years					
FVC (% of pred)	34	84.4 (2.2)	39	94.1 (1.4)	< 0.001
FEV_1_ (% of pred)	34	82.4 (2.4)	39	93.5 (1.5)	< 0.001
FEV_1_/FVC	34	90.4 (1.1)	39	91.8 (0.6)	0.256
cor. DLco (% of pred)	34	68.3 (1.9)	41	78.2 (1.8)	< 0.001
B. Boy, above 8 years					
FVC (% of pred)	23	83.1 (3.4)	52	98.5 (1.5)	< 0.001
FEV_1_ (% of pred)	23	81.7 (3.1)	52	95.9 (1.6)	< 0.001
FEV_1_/FVC	23	89.7 (1.0)	52	88.6 (0.6)	0.308
cor. DLco (% of pred)	28	74.4 (3.5)	55	89.3 (1.5)	< 0.001
Girl, above 8 years					
FVC (% of pred)	15	80.9 (2.3)	27	92.1 (1.5)	< 0.001
FEV_1_ (% of pred)	15	77.3 (2.7)	27	90.4 (1.4)	< 0.001
FEV_1_/FVC	15	89.8 (2.2)	27	91.5 (0.8)	0.465
cor. DLco (% of pred)	19	67.9 (2.0)	29	80.8 (2.3)	< 0.001
C. Boy, above 9 years					
FVC (% of pred)	18	81.0 (3.6)	31	98.1 (1.8)	< 0.001
FEV_1_ (% of pred)	18	79.3 (3.0)	31	95.1 (1.9)	< 0.001
FEV_1_/FVC	18	89.7 (1.1)	31	88.5 (0.7)	0.390
cor. DLco (% of pred)	25	74.1 (3.4)	32	89.6 (2.3)	< 0.001
Girl, above 9 years					
FVC (% of pred)	13	80.8 (2.6)	15	90.5 (1.7)	0.004
FEV_1_ (% of pred)	13	76.6 (3.0)	15	87.7 (1.4)	0.004
FEV_1_/FVC	13	89.2 (2.5)	15	91.3 (1.1)	0.462
cor. DLco (% of pred)	15	68.3 (2.1)	15	83.7 (2.6)	< 0.001

*P* values for the comparison between HDLI survivors and controls calculated with the Student t test. HDLI, HD associated lung injury; N, number; SE, standard errors of the estimated mean; pred, predicted; FVC, forced vital capacity; FEV_1_, forced expiratory volume in 1 sec; FEV_1_/FVC, the ratio of forced expiratory volume in 1 sec to forced vital capacity; FEF_25–75_: forced expiratory flow between 25 and 75% of vital capacity; DLco, diffusing capacity of the lung for carbon monoxide; cor. DLco, corrected DLco

### Longitudinal assessment of spirometry and DLco in HDLI survivors

**Fig. 1:**
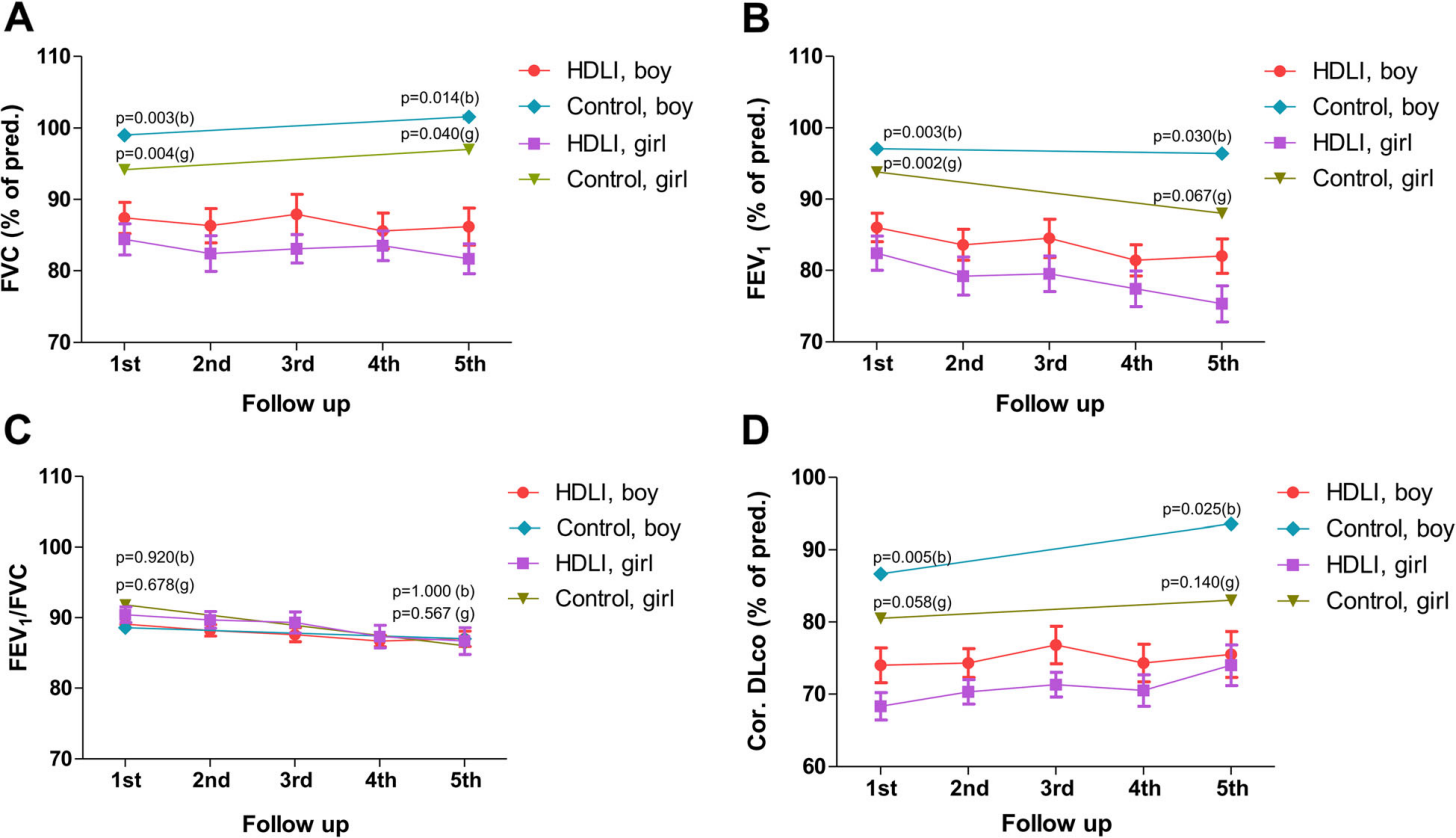
Longitudinal assessment of spirometry and DLco in HDLI survivors compared with control. Figures show the mean changes in FVC (**a**), FEV_1_ (**b**), FEV_1_/FVC (**c**), and corrected DLco (**d**) in the follow-up period, presented as the percent of the predicted values for HDLI survivors compared to controls. Controls were compared with age- and sex-matched HDLI children both at the 1st and 5th follow-up. *P*-values were calculated for the comparisons between groups for each sex using the Kruskall-Wallis test. Pred, predicted; FVC, forced vital capacity; FEV_1_, forced expiratory volume in 1 sec; Cor., corrected; DLco, carbon monoxide diffusion capacity; b, boy; g, girl

In the long-term growth characteristics of HDLI survivors and control subjects, there was no apparent difference in the final growth in both spirometry and DLco assessment in Additional file 3: Figure S2. The longitudinal assessment of lung function at the five time points in HDLI survivors compared with controls is shown in Fig. 1. The mean percent of predicted FVC in HDLI survivors of both sexes was within the normal range during the follow-up. However, FVC were not more than 90% in both sexes (in detail, mean percent of predicted value of FVC was 87.4% at 1st, 86.3% at 2nd, 87.9% at 3rd, 85.6% at 4th, and 86.2% at the 5th follow-up in boys and 84.4% at 1st, 82.4% at 2nd, 83.1% at 3rd, 83.5% at 4th, and 81.7% at the 5th follow-up in girls) and significantly lower compared with the control group in both sexes. Corrected DLco was persistently reduced to less than 80% in both sexes without recovery in HDLI survivors (boys, 74.0% in 1st, 74.3% in 2nd, 76.8% in 3rd, 74.3% in 4th, and 75.5% in the 5th follow-up; girls, 68.3% in 1st, 70.3% in 2nd, 71.3% in 3rd, 70.5% in 4th, and 74.0% in the 5th follow-up), compared with controls, especially boys. The mean percent of predicted FEV_1_ was also significantly lower compared with that of the control group. The ratio of FEV_1_/FVC remained relatively similar between HDLI survivors and controls within the normal range. These results also remained significant in FVC and corrected DLco of the z-scores from Global Lung Function Initiative (GLI) equations in Additional file 4: Figure S3 [25, 26].

**Fig. 2:**
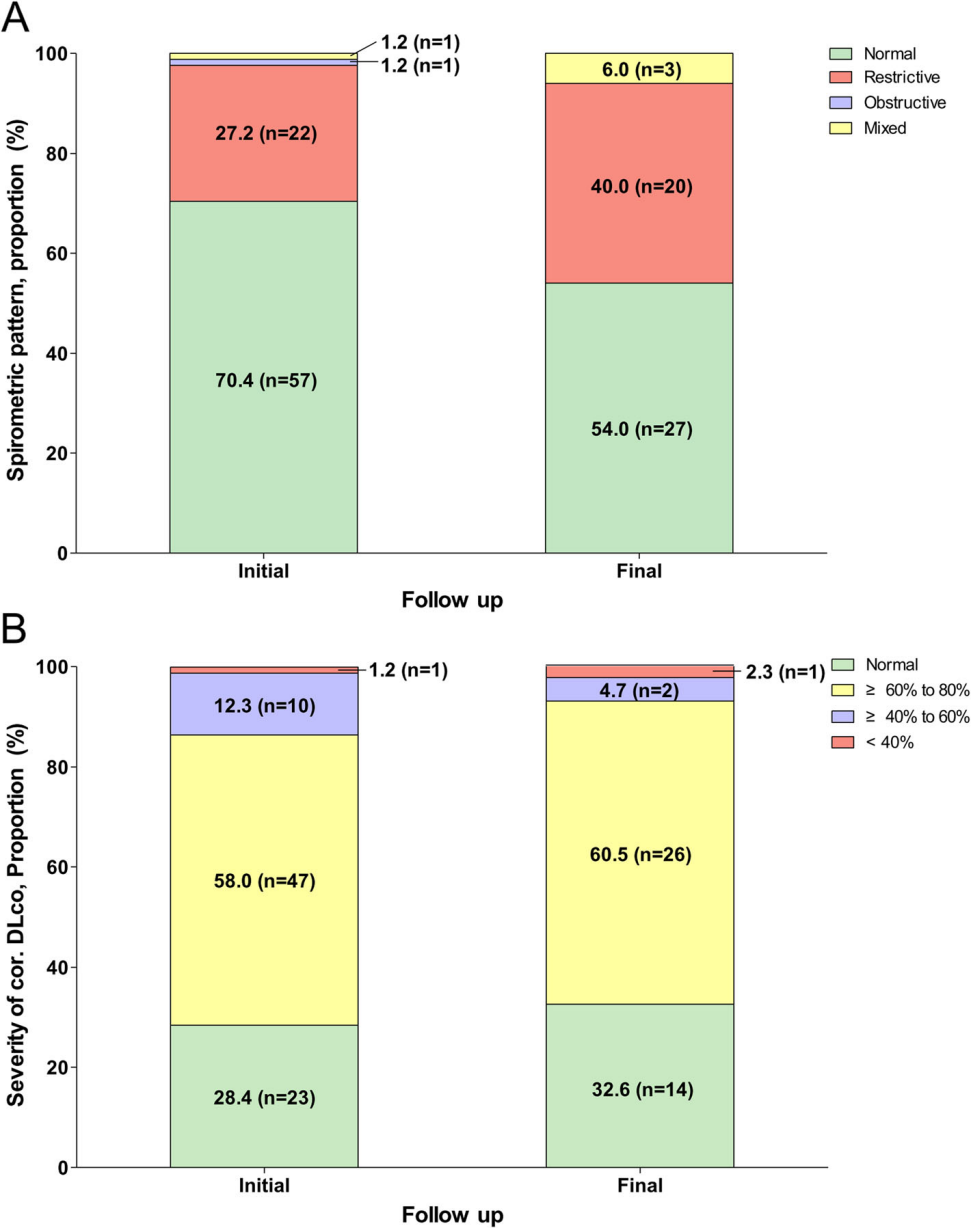
Spirometry pattern and DLco at initial and final follow-up in HDLI survivors. Figures show changes in the proportion of the spirometric pattern (**a**) and severity of corrected DLco (**b**) between the initial and final tests in HDLI survivors. In panel A, 81 and 50 children underwent spirometry at the 1st and 5th follow-ups, respectively. Participants showed the tendency to change to a restrictive pattern. In panel B, 81 and 43 children underwent DLco at the 1st and 5th follow-up, respectively. Most participants showed persistent decline in the diffusion defect, although some showed improvement

Additional analyses of spirometry abnormalities and severity of corrected DLco were also performed between the initial and final tests (Fig. 2). The prevalence of a restrictive pattern in both the initial and final tests was remarkable (27.2 and 40.0%, restrictively). In addition, the proportion of low corrected DLco for the initial test was 58 (71.5%) survivors, including 47 (58.0%) with mildly reduced DLco, 10 (12.3%) with moderated defect, and 1 (1.2%) with severe defect. At the final follow-up, 29 (67.5%) had abnormal DLco, with 26 (60.5%) mild, 2 (4.7%) moderate, and 1 (2.3%) severely low values.

### Lung function phenotype of HDLI survivor associated exposure characteristics

**Fig. 3:**
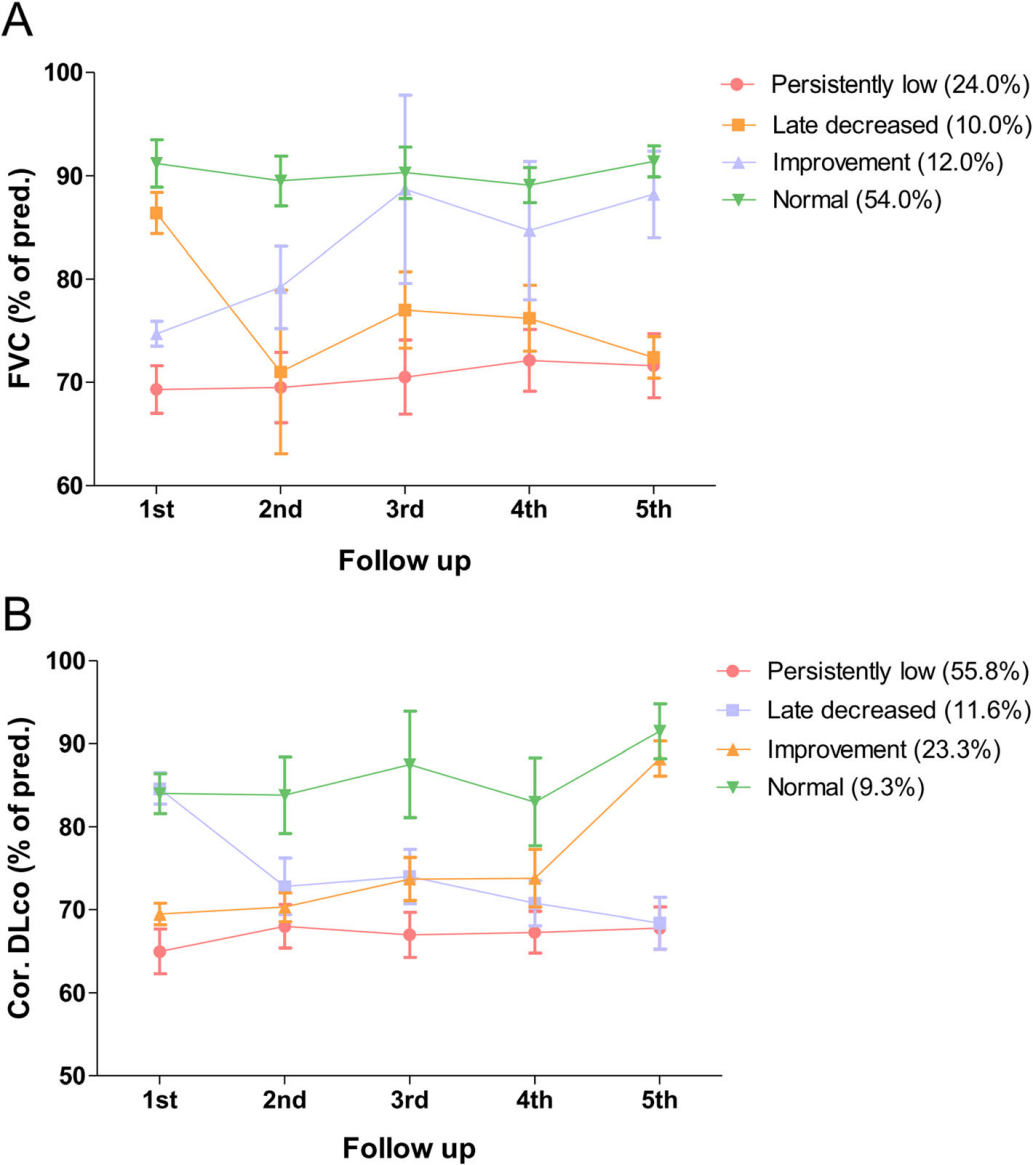
Prognosis phenotypes in corrected DLco and FVC over time by mean predicted %. Data for the prognosis of phenotypes in the follow-up period in HDLI survivors are shown. Considering the significant diffusion defect to be less than 80% at each follow-up, from the 1st to 5th, the proportion of each type of prognosis of HDLI is evaluated. HDLI children predominantly presented with abnormal lung function phenotypes (persistently low and late decreased) with respect to the corrected DLco (67.4%) and FVC (34.0%). Bars indicate standard errors of the estimated mean (persistently low and late decreased)

We classified the four prognosis phenotypes in FVC and corrected DLco by comparing the initial and final examination as follows: (1) persistently low, (2) late decreased, (3) improvement, and (4) normal (Fig. 3). As the late decreased and improvement phenotypes are mixed with normal values, we selected the two patterns, which are persistently low and normal, and compared the exposure characteristics between the two phenotypes (Table 3). Interestingly, most proportion of children with persistently low lung function in both FVC and corrected DLco has the initial exposure within the first year of the age with statistically significant difference compared with normal lung function (FVC, 83.3% vs. 37.0%, *P* = 0.008; corrected DLco, 62.5% vs. 0.0% *P* = 0.020, respectively). Among the children with exposure to HD during the first year of life, none were in the range of normal corrected DLco. These significances of initial exposure age were also observed in the comparison between the four prognostic phenotypes (persistently low, late decreased, improvement, and normal) in both FVC and corrected DLco (Additional file 1: Table S2). There is a higher proportion of children with a history of fetal exposure to HD who have a persistent decrement in lung function compared with continued normal PFT, although with no statistically significant difference (FVC, 16.7% vs. 3.7%, *P* = 0.161; corrected DLco, 8.3% vs. 0.0%, *P* = 0.549, respectively).

**Table 3:** Comparison of exposure characteristics based on lung function phenotype in HDLI survivors

	FVC			Corrected DLco		
	Persistently low (*n* = 12)	Normal (*n* = 27)	p-value	Persistently low (*n* = 24)	Normal (*n* = 4)	p-value
HD type (%)						
PHMG, only use	50.0	51.9	0.787	50.0	25.0	0.353
PGH, only use	0.0	3.7		0	0	
CMIT/MIT, only use	0.0	3.7		0	0	
Mixed	50.0	40.7		50.0	75.0	
^a^HD exposure intensity/weight at exposure (%)						
1st quartile	16.7	33.3	0.749	29.2	25.0	0.564
2nd quartile	33.3	25.9		20.8	50.0	
3rd quartile	33.3	29.6		29.2	25.0	
4th quartile	16.7	11.1		20.8	0.0	
^a^HD exposure intensity during sleep/weight at exposure (%)						
1st quartile	25.0	29.6	0.509	33.3	25.0	0.037
2nd quartile	25.0	33.3		12.5	75.0	
3rd quartile	25.0	29.6		33.3	0.0	
4th quartile	25.0	7.4		20.8	0.0	
Age on initial exposure (%)						
< 12.0 months	83.3	37.0	0.008	62.5	0.0	0.020
≥ 12.0 months	16.7	63.0		37.5	100.0	
Fetal exposure (%)	16.7	3.7	0.161	8.3	0.0	0.549
Total duration of use (month)	13.7	15.9	0.690	15.7	22.9	0.466
Distance between bed and HD (%)						
< 1.0 m	50.0	44.5	0.748	54.2	0.0	0.044
≥ 1.0 m	50.0	55.6		45.8	100.0	
Direction dispersed into room (%)						
Diagonal	41.7	44.4	0.736	37.5	25.0	0.459
Forward	33.3	40.7		41.7	25.0	
Unknown	25.0	14.8		20.8	50.0	
Use of HD during sleep (%)	91.7	100.0	0.129	95.8	100.0	0.678

*P* values for the comparison between persistently low and normal calculated with the Chi-square or Student t-test, as appropriate, in FVC and corrected DLco. ^a^Airborne disinfectant intensity = [bulk level of disinfectant (μg/mL) × disinfectant volume (mL) × disinfectant frequency/day × room ventilation factor]/room volume (m^3^)/weight at exposure [23, 27]. FVC, forced vital capacity; DLco, diffusing capacity of the lung for carbon monoxide; N, number; HD, humidifier disinfectants; PHMG, polyhexamethyleneguanidine phosphate; PGH, poligoethoxyethyl-guanidinium chloride; CMIT/MIT, chloromethylisothiazol/methylisothiazol

The airborne HD exposure intensity showed no statistical difference between persistently low lung function and normal. However, airborne HD exposure intensity during sleep among the persistently low cases had a high proportion of 3rd and 4th quartile compared with normal cases, showing a statistically significant difference in corrected DLco (FVC, 50.0% vs. 37.0%, *P* = 0.509; corrected DLco, 54.1% vs. 0.0%, *P* = 0.037, respectively). Differences in the distance from the humidifier to the sleeping place also showed a statistically significant difference in persistently low corrected DLco. Half of the cases of persistently low corrected DLco were exposure within 1.0 m, whereas all the subjects of the normal corrected DLco were exposed to HD beyond 1.0 m. Neither HD type, total months, nor direction was associated with abnormal lung function during long-term follow-up. The Cox proportional hazards model for examining the association between age on initial exposure and lung function phenotype showed that exposure during the first year of life significantly increased the hazard ratio (HR) for persistently low corrected DLco [HR 1.030 (95% CI 1.001–1.060), *P* = 0.044] (Additional file 5: Figure S4A), but was not associated with the HR for persistently low FVC (Additional file 5: Figure S4B).

## Discussion

Lung function including FVC, FEV_1_, DLco, and corrected DLco were markedly decreased in HDLI survivors compared with controls in the cross-sectional study. In the longitudinal assessment, FVC was found to be consistently low despite being within the normal range, and the corrected DLco was found to have no normalization over 80% with increasing age, although no apparent difference was seen in final growth between HDLI survivors and controls. Children with exposure within the first year of life have been associated with persistently low lung function, suggesting a critical period related to airborne chemicals. Notably, initial exposure age under 1 year increased the hazard risk of persistent decrement in corrected DLco adjusted for height and weight at DLco performance, HD type, and total months of use. These findings suggest that we may consider prolonged decrement in corrected DLco as a valuable marker of lung involvement in children with a history of HD exposure. Furthermore, persistent abnormalities with corrected DLco are also associated with higher intensity HD exposure during sleep and close distance between the bed and the humidifier. As most subjects were exposed during sleep, the difference in airborne disinfectant exposure intensity during sleep is believed to be significant.

To the best of our knowledge, this analysis is the first to address lung function impairment and the phenotype-associated sensitive window in relation to HD inhalation exposure in children with a combination of case–control and cohort designs. In addition, we confirmed the first longitudinal lung impairment in children by chemical inhalants and may provide guidance on evaluating lung injury caused by chemical inhalation.

Many recent studies have reported that environmental exposure in early-life affects the long-term prognosis of respiratory disease during childhood [28, 29]. Furthermore, identification of the critical exposure window can suggest more effective interventions for lung development during childhood [30–32]. The present study has addressed these concerns using the association between lung function and the use of household chemical disinfectant. This study highlights the need for further evaluation and continued follow-up of pulmonary function in children with chemical inhalation during the critical period, regardless of the duration following the exposure.

The mechanism by which HD exposure affects prolonged decrement in lung function, especially corrected DLco among children who are exposed within the first year of life, remains unknown. Possibilities include that HDs were dissolved in water and then dispersed into the air by the humidifier’s aerosolizer, and the nano-sized particles allowed them to easily reach the distal airways causing lung injury. HDLI with pathologic findings such as bronchiolar destruction and obliteration, centrilobular alveolar damage, and remodeling by inflammatory may support the above-mentioned evidence [10, 11]. Respiratory inhalational toxicants including irritant gases or sensitizers have also been investigated to cause acute inhalation injury by tissue asphyxiation including inhibition of mitochondrial electron transport and oxygen use or direct airway cellular injury [33]. Additionally, in the first 2 years of life, the alveolar volume increases with age and somatic growth [24]. High density and close exposure to HD during sleep within the first year of life causes accelerated lung injury and disruption of alveolar development, resulting in lower corrected DLco. As the bronchioles, alveolar ducts, and alveolus functionally mature at approximately 2 years of age, alterations in these tissues could result in inappropriate distal lung development, resulting in restrictive lung disease. An additional explanation of the association between lung function in children and HD exposure in the first year of life [34] is that infants may spend more time at home and may be at a high risk because they take in more of the contaminant than adults relative to their body size, and have particularly vulnerable physiologies, as the minute ventilation per pound in an infant is significantly greater than that of an adult [35]. Particularly, children breath 50% more air per kg of weight compared to adults, and their habits are conducive to greater exposures [36]. As an infant’s room is in a relatively confined space, and infants cannot change positions by themselves during sleep, this can result in considerable cumulative exposure during sleep and association between corrected DLco and HD exposure intensity during sleep than the usual HD exposure intensity in our study. These results are consistent with those of a previous report on impulse oscillometry abnormality in HD-exposed children [37]. Further investigation is required to identify the pathophysiological mechanisms of this association.

Furthermore, a high proportion of children with a history of fetal exposure to HD showed persistent decrement in lung function, although without statistical significance, due to the small sample size. The placenta plays a role in exchanging foreign molecules between the maternal and fetal circulations, suggesting that HD may cause developmental toxicity in the fetuses [38]. Future studies are required to investigate whether HD chemicals cross the placenta and accumulate in the fetal tissues.

To date, no longitudinal research on DLco in children has investigated the pathophysiology of chemical airborne lung injury. DLco is an outcome of particular interest, as exposure related lung function in asymptomatic children without a chronic respiratory disease because exposure to respiratory irritants is known to cause small airway disease and affect transfer capability [39]. Impairment recovery of corrected DLco in HD-exposed children, and association between exposure characteristics and the corrected DLco outcome in our study may support the above-mentioned evidence. These findings suggest that the corrected DLco seems to be more sensitive for detecting lung damage as one of the alternative biomarkers of children with HDLI and as a long-lasting indicator in lung injury after exposure to HD in children. As approximately 30% of young Korean children were exposed to HD, although without any obvious clinical or radiologic findings, this reflects the importance of corrected DLco in the detection of latent respiratory effects in children [40]. The measurement of DLco in children is under investigation, with no generally available apparatus, and we may be unable to detect potential lung damage in many children. Therefore, in cases of absence of risk factors (including chronic respiratory disease), other than the exposure to HD in early-life, the persistent decrement in corrected DLco in Korean school children may imply an adverse impact of HD exposure on the lung function.

The strengths of the present study are that this is a well-characterized, relatively large HDLI cohort study. The PFT result was also provided by highly skilled leaders using a fixed standard operating procedure in a pediatric pulmonology laboratory. Second, the study participants are purely control subjects, in which there was no exposure to HD compared other studies about exposure to air pollution and other household chemicals. Furthermore, as a lower prevalence of chronic respiratory conditions, including smoking history, was reported in children than in adults, HDLI survivors were considered to be a pure disease group. Therefore, we may suggest adequate data to make conclusions about the HD exposure-lung function relationship.

We are aware that this study has limitations. First, we did not perform spirometry in the event because of too young children and severely affected patients (expired or not performed), and thus we cannot determine whether for some patients there was an even more severe immediate decrease in lung function and subsequent incomplete recovery. This may have resulted in a weaker exposure-response relationship. Second, there could be a risk of recall bias because most characteristics on disinfectant use were obtained from a direct questionnaire given to the study subjects. However, it is fairly reliable, considering that previous studies have already reported HDLI characteristics using this exposure data [15, 23]. Third, we could not explore the effects of exposure from adolescence to young adulthood, and the long-term clinical significance with respect to lung function is still unknown. Considering the lung growth prognosis of children exposed to HD, a longer follow-up is required to investigate whether children with HDLI with an early exposure will catch up in lung growth to peak lung function in adolescence and adulthood or will continue to have a decline in lung function [41].

## Conclusions

In conclusion, HD inhalation exposure within the first year of life may constitute a risk to respiratory health not only in terms of HDLI, but also as a long-term impact resulting in decreased lung function in children. Although the disease-specific biomarkers remain unclear, periodic evaluation of the children using PFTs will provide a unique opportunity to follow disease progression and to identify alternative diagnostic markers for children with HDLI.

## Supplementary information

**Additional file 1: Table S1.** Chemical airborne exposure-related lung function. **Table S2.** Comparison of characteristics and exposure index based on 4 lung function phenotype (FVC (A) and corrected DLco (B)) in HDLI survivors.

**Additional file 2: Figure S1*.*** HDLI patients and control enrollment flow diagram.

**Additional file 3: Figure S2*.*** Longitudinal assessment of age and height.

**Additional file 4: Figure S3.** Longitudinal assessment in HDLI vs. control subjects for z-score.

**Additional file 5: Figure S4.** Cox proportional hazard regression model showing the effect of exposure periods on persistently low corrected DLco.

## Data Availability

The datasets used and/or analyzed during the current study are available from the corresponding author on reasonable request.

## Abbreviations

chILD: Childhood interstitial lung disease
DLco: Diffusing capacity of the lung for carbon monoxide
FEV_1_: Forced expiratory volume in 1 s
FEV_1_/FVC: The ratio of forced expiratory volume in 1 sec to forced vital capacity
FVC: Forced vital capacity
HD: Humidifier disinfectants
HDLI: Humidifier disinfectant associated lung injury
PFT: Pulmonary function test
