# Supplementary material for: Early-life exposure to humidifier disinfectant determines the prognosis of lung function in children
Source: BMC Pulm Med. 2019 Dec 23;19:261. doi: 10.1186/s12890-019-1028-y (PMC6929365; doi:10.1186/s12890-019-1028-y)
Supplement: Supplementary file 1 — Additional file 1: Table S1. Chemical airborne exposure-related lung function. Table S2. Comparison of characteristics and exposure index based on 4 lung function phenotype (FVC (A) and corrected DLco (B)) in HDLI survivors. [file 12890_2019_1028_MOESM1_ESM.doc]

**Additional files**

**Early-life exposure to humidifier disinfectant determines the prognosis of lung function in children**

Hyun-Ju Cho, MD^1^, So Yeon Lee, MD, PhD^2^, Donguk Park, PhD^3^, Seung-Hun Ryu, PhD^4^, Jisun Yoon, MD^5^, Sungsu Jung, MD^6^, Eun Lee, MD, PhD^7^, Song-I Yang, MD, PhD^8^, Soo-Jong Hong, MD, PhD^2^*

^1^Department of Pediatrics, International St. Mary's hospital, Catholic Kwandong University College of Medicine, Incheon, Republic of Korea, ^2^Department of Pediatrics, Childhood Asthma Atopy Center, Environmental Health Center, Asan Medical Center, University of Ulsan College of Medicine, Seoul, Republic of Korea, ^3^Department of Environmental Health, Korea National Open University, Seoul, Republic of Korea, ^4^Department of Environmental Health Sciences, Graduate School of Public Health, Seoul National University, Seoul, Republic of Korea, ^5^Department of Pediatrics, MediplexSejong Hospital, Republic of Korea,

^6^Department of Pediatrics, Pusan National University Yangsan Hospital, Yangsan, Republic of Korea, ^7^Department of Pediatrics, Chonnam National University Hospital, Chonnam National University Medical School, Gwangju, Republic of Korea, ^8^Department of Pediatrics, Hallym University Sacred Heart Hospital, Hallym University College of Medicine, Anyang, Korea

***Table S1.*** ***Chemical airborne exposure-related lung function***

| **Exposure** | **Location** | **Study design** | **Illustrative setting** | **Spirometry** | **Predominantly**  **Pattern** | **Ref.** |
| --- | --- | --- | --- | --- | --- | --- |
| VOCs | Iran | Case-control,  40 adults | Automobile manufacturing | ↓ FVC | NA | [1] |
| TDI | USA | Prospective,  197 adults | Production workers | ↓FEV_1_, FVC | Restrictive | [2] |
|  | USA | Prospective,  49 adults | Polyurethane foam factory | ↓FEV_1_ | Obstructive | [3] |
| HD | South Korea | Prospective,  40 adults | Home | ↓ FVC | Restrictive | [4] |
|  | South Korea | Cross-sectional,  17 adults | Home | ↓ FVC (&DLco) | Restrictive | [5] |
|  | South Korea | Prospective,  81 children | Home | ↓ FVC (&DLco) | Restrictive | Current |
| SAS | Germany | Cross-sectional, 462 adults | Production workers | ↓ FVC | Obstructive (17%), Restrictive (4%) | [6] |
| Chlorine gas | USA | Prospective, 1807, adults | Train derailment | ↓FEV_1_ | NA | [7] |
|  | USA | Cross-sectional,  22 children,  198 adults | Train derailment | ↓FEV_1_ | Restrictive (36%) | [8] |
| Diacetyl | USA | Prospective,  106 adults | Manufacturing workers | ↓FEV_1_, FVC | Restrictive | [9] |
| Welding fume | Iran | Cross-sectional, 43 adults | Automobile assembly welders | ↓ FEF_25-75_ | NA | [10] |
| Sulfur mustard | Iran | Prospective,  479 adults | Iraq–Iran war victims | ↓FEV_1_ | NA | [11] |
|  | Iran | Retrospective,  603 adults | Iraq–Iran war victims | NA | Obstructive | [12] |
|  | Iran | Prospective  220 adults | Iraq–Iran war victims | NA | Obstructive | [13] |
| Crude oil | Saudi Arabia | Cross-sectional,  20 adults | Spill in sea water | ↓FEV_1_, FVC, FEF_25-75_ | NA | [14] |
| Ammonia | Bangkok | Cross-sectional,  88 adults | Urea fertilizer factory | ↓FEV_1_, FVC | NA | [15] |
|  | Saudi Arabia | Cross-sectional,  77 adults | Ammonia factory | ↓FEV_1_, FVC | mixed | [16] |
| Cadmium | India | Cross-sectional, 133 adults | Jewelry workshop | ↓FEV_1_, FVC | NA | [17] |
|  | Poland | Cross-sectional. 79 adults | Battery workers | ↓ FEV_1_, FEF_25-75_ | Obstructive | [18] |
| Bhopal gas  (methyl isocyanate gas) | UK | Cross-sectional,  30 adults | Residence | ↓ FEF_25-75_ | Obstructive | [19] |
|  | India | Cross-sectional,  454 adults | Residence | ↓FEV_1_, FVC, FEV1/FVC | Obstructive | [20] |
| Aluminum | Turkey | Cross-sectional,  55 adults | Factory | ↓FEV_1_, FVC, FEV_1_/FVC (&DLco) | NA | [21] |

References for this review were identified through searches of PubMed for articles published from January 1971 to August 2018, by use of the terms “chemical” and “spirometry”, “chemical”, and “diffusing capacity”. Articles published in English were included. NA, not available. VOCs, Volatile organic compound; TDI, Toluene Diisocyanate; HD, Humidifier disinfectant; SAS, Synthetic Amorphous Silica

**Text S1. Exposure Assessment to HD**

The Lung Injury Investigation Committee (LIIC) also consisted of experts in occupational and environmental hygiene, who took part in the home exposure assessments. The exposure assessment team was blinded to the study subjects’ clinically diagnosed lung disease information. Three trained environmental health scientists conducted both a home investigation and a personal interview related to the use of HDs and demographic characteristics. We asked the study participants, or in the case of children, their parents or guardians, to complete a detailed questionnaire collecting information related to HD use. To aid in recall, participants were shown photographic examples of all the HD products that had been marketed in South Korea. Home visits and administration of a standardized questionnaire were arranged to assess exposure circumstances, including the following: whether any HD remained in the home; daily and seasonal patterns of humidifier and HD use; the name, amount, and frequency of HD added to the humidifier water tank; room size and ventilation; and the general condition of each home. Details of exposures of every household family member were determined so that those who were exposed but had not developed any symptoms or other evidence of lung disease were identified in the same way as those who were exposed and had developed symptoms or disease. Family members who were probably not exposed were also identified.

The likelihood of exposure of individuals was first ascertained based on objective evidence of remaining disinfectant in the household or consistent and detailed parent statements on the purchase and use of HD products. Airborne HD exposure intensity (C, µg/m^3^) was calculated based on the bulk level of HD, the total HD volume (mL), hours of use per day (h), room size in which the HD was used (m^3^) and assumed ventilation rate. Air change per hour (ACH) in the room was assumed to be 0.5. The ventilation rate (Q, m^3^/h) was calculated based on the room size and an ACH of 0.5. Finally, airborne HD exposure intensity was calculated by multiplying by C and Q, and this was divided by weight at exposure to adjust for differences in somatic size among subjects [22].

**Reference**

1. Harati B, Shahtaheri SJ, Karimi A, Azam K, Ahmadi A, Afzali Rad M, Harati A: **Evaluation of Respiratory Symptoms among Workers in an Automobile Manufacturing Factory, Iran**. *Iranian journal of public health* 2018, **47**(2):237-245.

2. Wang ML, Storey E, Cassidy LD, Doney B, Conner PR, Collins JJ, Carson M, Molenaar D: **Longitudinal and Cross-sectional Analyses of Lung Function in Toluene Diisocyanate Production Workers**. *J Occup Environ Med* 2017, **59 Suppl 12**:S28-s35.

3. Gui W, Wisnewski AV, Neamtiu I, Gurzau E, Sparer JA, Stowe MH, Liu J, Slade MD, Rusu OA, Redlich CA: **Inception cohort study of workers exposed to toluene diisocyanate at a polyurethane foam factory: initial one-year follow-up**. *American journal of industrial medicine* 2014, **57**(11):1207-1215.

4. Kim WY, Park S, Kim HJ, Chae EJ, Do KH, Huh JW, Lim CM, Koh Y, Hong SB: **Lung function in patients with lung injury due to household chemical inhalation: Post hoc analysis of a prospective nationwide cohort**. *Respirology (Carlton, Vic)* 2017, **22**(2):345-351.

5. Hong SB, Kim HJ, Huh JW, Do KH, Jang SJ, Song JS, Choi SJ, Heo Y, Kim YB, Lim CM *et al*: **A cluster of lung injury associated with home humidifier use: clinical, radiological and pathological description of a new syndrome**. *Thorax* 2014, **69**(8):694-702.

6. Taeger D, McCunney R, Bailer U, Barthel K, Kupper U, Bruning T, Morfeld P, Merget R: **Cross-Sectional Study on Nonmalignant Respiratory Morbidity due to Exposure to Synthetic Amorphous Silica**. *J Occup Environ Med* 2016, **58**(4):376-384.

7. Clark KA, Karmaus WJ, Mohr LC, Cai B, Balte P, Gibson JJ, Ownby D, Lawson AB, Vena JE, Svendsen ER: **Lung Function before and after a Large Chlorine Gas Release in Graniteville, South Carolina**. *Annals of the American Thoracic Society* 2016, **13**(3):356-363.

8. Clark KA, Chanda D, Balte P, Karmaus WJ, Cai B, Vena J, Lawson AB, Mohr LC, Gibson JJ, Svendsen ER: **Respiratory symptoms and lung function 8-10 months after community exposure to chlorine gas: a public health intervention and cross-sectional analysis**. *BMC public health* 2013, **13**:945.

9. Kreiss K: **Work-related spirometric restriction in flavoring manufacturing workers**. *American journal of industrial medicine* 2014, **57**(2):129-137.

10. Sharifian SA, Loukzadeh Z, Shojaoddiny-Ardekani A, Aminian O: **Pulmonary adverse effects of welding fume in automobile assembly welders**. *Acta medica Iranica* 2011, **49**(2):98-102.

11. Ghanei M, Naderi M, Kosar AM, Harandi AA, Hopkinson NS, Poursaleh Z: **Long-term pulmonary complications of chemical warfare agent exposure in Iraqi Kurdish civilians**. *Inhalation toxicology* 2010, **22**(9):719-724.

12. Ghanei M, Adibi I, Farhat F, Aslani J: **Late respiratory effects of sulfur mustard: how is the early symptoms severity involved?** *Chronic respiratory disease* 2008, **5**(2):95-100.

13. Bijani K, Moghadamnia AA: **Long-term effects of chemical weapons on respiratory tract in Iraq-Iran war victims living in Babol (North of Iran)**. *Ecotoxicology and environmental safety* 2002, **53**(3):422-424.

14. Meo SA, Al-Drees AM, Meo IM, Al-Saadi MM, Azeem MA: **Lung function in subjects exposed to crude oil spill into sea water**. *Marine pollution bulletin* 2008, **56**(1):88-94.

15. Rahman MH, Bratveit M, Moen BE: **Exposure to ammonia and acute respiratory effects in a urea fertilizer factory**. *International journal of occupational and environmental health* 2007, **13**(2):153-159.

16. Ali BA, Ahmed HO, Ballal SG, Albar AA: **Pulmonary function of workers exposed to ammonia: a study in the Eastern Province of Saudi Arabia**. *International journal of occupational and environmental health* 2001, **7**(1):19-22.

17. Moitra S, Blanc PD, Sahu S: **Adverse respiratory effects associated with cadmium exposure in small-scale jewellery workshops in India**. *Thorax* 2013, **68**(6):565-570.

18. Jakubowski M, Abramowska-Guzik A, Szymczak W, Trzcinka-Ochocka M: **Influence of long-term occupational exposure to cadmium on lung function tests results**. *International journal of occupational medicine and environmental health* 2004, **17**(3):361-368.

19. Dhara VR, Dhara R, Acquilla SD, Cullinan P: **Personal exposure and long-term health effects in survivors of the union carbide disaster at bhopal**. *Environmental health perspectives* 2002, **110**(5):487-500.

20. Cullinan P, Acquilla S, Dhara VR: **Respiratory morbidity 10 years after the Union Carbide gas leak at Bhopal: a cross sectional survey. The International Medical Commission on Bhopal**. *BMJ (Clinical research ed)* 1997, **314**(7077):338-342.

21. San LN, Uysal H, Gokbel H, Bediz CS, Sayal A: **Pulmonary function of workers in the aluminum industry**. *American journal of industrial medicine* 1998, **33**(3):305-307.

22. Williams PR, Patterson J, Briggs DW: **VCCEP pilot: progress on evaluating children's risks and data needs**. *Risk analysis : an official publication of the Society for Risk Analysis* 2006, **26**(3):781-801.

23. Park DU, Friesen MC, Roh HS, Choi YY, Ahn JJ, Lim HK, Kim SK, Koh DH, Jung HJ, Lee JH *et al*: **Estimating retrospective exposure of household humidifier disinfectants**. *Indoor air* 2015, **25**(6):631-640.

**Table S2. Comparison of characteristics and exposure index based on 4 lung function phenotype (FVC (A) and corrected DLco (B)) in HDLI survivors**

|  | FVC (A) | | | | |
| --- | --- | --- | --- | --- | --- |
|  | Persistently low (n=12) | Late  Decreased (n=5) | Improvement  (n=6) | Normal  (n=27) | p-value |
| HD type (%)  PHMG, only use  PGH, only use  CMIT/MIT, only use  Mixed | 50.0  0.0  0.0  50.0 | 20.0  0.0  0.0  80.0 | 66.7  0.0  0.0  33.3 | 51.9  3.7  3.7  40.7 | 0.866 |
| ^‡^HD exposure intensity (µg/m^3^)  /weight at exposure (%)  1^st^ quartile  2^nd^ quartile  3^rd^ quartile  4^th^ quartile | 16.7  33.3  33.3  16.7 | 20.0  20.0  60.0  0.0 | 50.0  33.3  0.0  16.7 | 33.3  25.9  29.6  11.1 | 0.699 |
| ^‡^HD exposure intensity during sleep (µg/m^3^) /weight at exposure (%)  1^st^ quartile  2^nd^ quartile  3^rd^ quartile  4^th^ quartile | 25.0  25.0  25.0  25.0 | 20.0  20.0  60.0  0.0 | 50.0  33.3  0.0  16.7 | 29.6  33.3  29.6  7.4 | 0.544 |
| Age on initial exposure (%)  < 12.0 months  ≥12.0 months | 83.3  16.7 | 80.0  20.0 | 50.0  50.0 | 37.0  63.0 | 0.034 |
| Fetal exposure (%) | 16.7 | 0.0 | 16.7 | 3.7 | 0.400 |
| Total months of use (month) | 13.7 | 22.8 | 22.4 | 15.9 | 0.614 |
| Distance between the bed and HD (%)  <1.0 m  ≥1.0 m | 50.0  50.0 | 60.0  40.0 | 16.7  83.3 | 44.4  55.6 | 0.472 |
| Direction dispersed into room (%)  Diagonal  Forward  Unknown | 41.7  33.3  25.0 | 20.0  80.0  0.0 | 66.7  16.7  16.7 | 44.4  40.7  14.8 | 0.451 |
| Use of HD during sleep (%) | 91.7 | 100.0 | 100.0 | 100.0 | 0.357 |

|  | Corrected DLco (B) | | | | | |
| --- | --- | --- | --- | --- | --- | --- |
|  | Persistently low (n=24) | | Late  Decreased (n=5) | Improvement (n=10) | Normal (n=4) | p-value |
| HD type (%)  PHMG, only use  PGH, only use  CMIT/MIT, only use  Mixed | 50.0  0.0  0.0  50.0 | 80.0  0.0  0.0  .20.0 | | 60.0  0.0  10.0  30.0 | 25.0  0.0  0.0  75.0 | 0.334 |
| ^‡^HD exposure intensity (µg/m^3^)  /weight at exposure (%)  1^st^ quartile  2^nd^ quartile  3^rd^ quartile  4^th^ quartile | 29.2  20.8  29.2  20.8 | 0.0  60.0  40.0  0.0 | | 30.0  30.0  30.0  10.0 | 25.0  50.0  25.0  0.0 | 0.684 |
| ^‡^HD exposure intensity during sleep (µg/m^3^) /weight at exposure (%)  1^st^ quartile  2^nd^ quartile  3^rd^ quartile  4^th^ quartile | 33.3  12.5  33.3  20.8 | 0.0  80.0  20.0  0.0 | | 30.0  30.0  30.0  10.0 | 25.0  75.0  0.0  0.0 | 0.103 |
| Age on initial exposure (%)  < 12.0 month  ≥12.0 month | 62.5  37.5 | 40.0  60.0 | | 70.0  30.0 | 0.0  100.0 | 0.078 |
| Fetal exposure (%) | 8.3 | 0.0 | | 20.0 | 0.0 | 0.635 |
| Total months of use (month) | 15.7 | 12.5 | | 19.0 | 22.9 | 0.790 |
| Distance between the bed and HD (%)  <1.0 m  ≥1.0 m | 54.2  45.8 | 40.0  60.0 | | 40.0  60.0 | 0.0  100.0 | 0.236 |
| Direction dispersed into room (%)  Diagonal  Forward  Unknown | 37.5  41.7  20.8 | 60.0  40.0  0.0 | | 60.0  40.0  0.0 | 25.0  25.0  50.0 | 0.302 |
| Use of HD during sleep (%) | 95.8 | 100.0 | | 100.0 | 100.0 | 0.847 |

P values for the comparison between persistently low and normal calculated with the Chi-square or Student t-test, as appropriate, in FVC and corrected DLco. ^a^Airborne disinfectant intensity = [bulk level of disinfectant (μg/mL) × disinfectant volume (mL) × disinfectant frequency/day × room ventilation factor]/room volume (m^3^)/weight at exposure [22, 23]. FVC, forced vital capacity; DLco, diffusing capacity of the lung for carbon monoxide; N, number; HD, humidifier disinfectants; PHMG, polyhexamethyleneguanidine phosphate; PGH, poligoethoxyethyl-guanidinium chloride; CMIT/MIT, chloromethylisothiazol/methylisothiazol
