# Supplementary figures and images for: Early-life exposure to humidifier disinfectant determines the prognosis of lung function in children
Source: BMC Pulm Med. 2019 Dec 23;19:261. doi: 10.1186/s12890-019-1028-y (PMC6929365; doi:10.1186/s12890-019-1028-y)

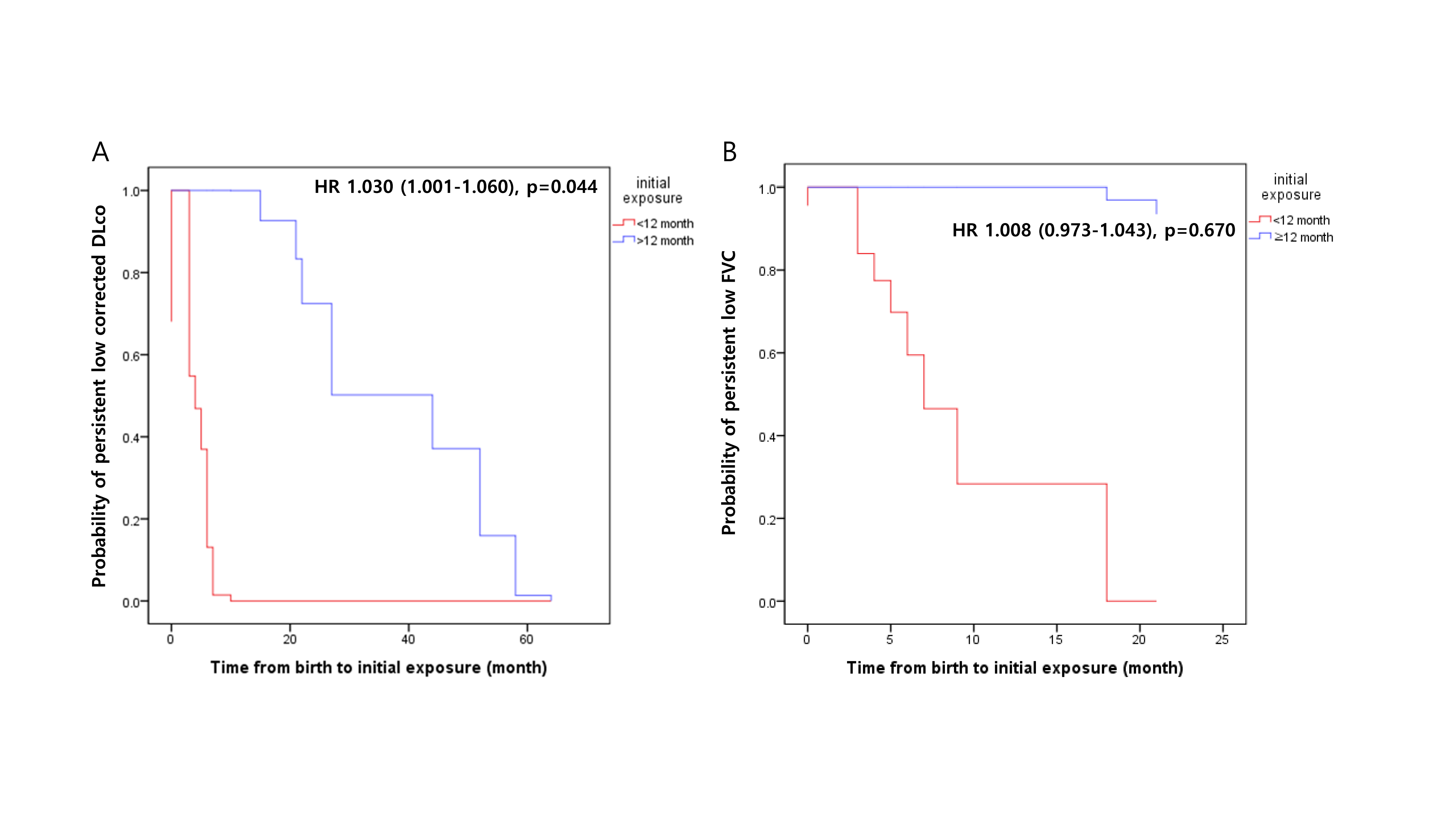

Supplement: Supplementary file 5 — Additional file 5: Figure S4. Cox proportional hazard regression model showing the effect of exposure periods on persistently low corrected DLco. [file 12890_2019_1028_MOESM5_ESM.tif]
